# Acute Psychosis as Main Manifestation of Central Pontine Myelinolysis

**DOI:** 10.1155/2017/1471096

**Published:** 2017-03-14

**Authors:** Mangala Gopal, Melvin Parasram, Harsh Patel, Chike Ilorah, Hrachya Nersesyan

**Affiliations:** ^1^College of Osteopathic Medicine, Des Moines University, Des Moines, IA, USA; ^2^Arizona College of Osteopathic Medicine, Midwestern University, Glendale, AZ, USA; ^3^Baroda Medical College, Vadodara, Gujarat, India; ^4^Department of Neurology, University of Illinois College of Medicine at Peoria, Peoria, IL, USA; ^5^Illinois Neurological Institute, OSF St. Francis Medical Center, Peoria, IL, USA

## Abstract

Central pontine myelinolysis (CPM) is an acute demyelinating neurological disorder affecting primarily the central pons and is frequently associated with rapid correction of hyponatremia. Common clinical manifestations of CPM include spastic quadriparesis, dysarthria, pseudobulbar palsy, and encephalopathy of various degrees; however, coma, “locked-in” syndrome, or death can occur in most severe cases. Rarely, CPM presents with neuropsychiatric manifestations, such as personality changes, acute psychosis, paranoia, hallucinations, or catatonia, typically associated with additional injury to the brain, described as extrapontine myelinolysis (EPM). We present a patient with primarily neuropsychiatric manifestations of CPM, in the absence of focal neurologic deficits or radiographic extrapontine involvement. A 51-year-old female without significant medical history presented with dizziness, frequent falls, diarrhea, generalized weakness, and weight loss. Physical examination showed no focal neurological deficits. Laboratory data showed severe hyponatremia, which was corrected rather rapidly. Subsequently, the patient developed symptoms of an acute psychotic illness. Initial brain magnetic resonance imaging (MRI) was unremarkable, although a repeat MRI two weeks later revealed changes compatible with CPM. This case demonstrates that acute psychosis might represent the main manifestation of CPM, especially in early stages of the disease, which should be taken into consideration when assessing patients with acute abnormalities of sodium metabolism.

## 1. Introduction

Central pontine myelinolysis (CPM) is a rare neurological syndrome first described in 1959 by Adams et al. [1]. It is an acute demyelinating condition primarily affecting central pons and commonly presenting clinically with spastic quadriparesis, dysarthria, pseudobulbar palsy, and altered mental status. In some patients, parkinsonian features, behavioral manifestations, and neuropsychological symptoms can also be present [2–5]. Pathophysiology of CPM consists of osmotic demyelination in the central pons with relative sparing of axons and neurons and is commonly associated with chronic alcoholism, liver failure, severe burns, malignant neoplasms, hemorrhagic pancreatitis, hemodialysis, and sepsis [6–8]. Rapid correction of hyponatremia has been proven as one of the most common and important etiologic factors [9, 10]. Although central pontine and extrapontine myelinolysis can present with behavioral and neuropsychiatric manifestations, there is limited literature available describing behavioral manifestations (personality changes, labile affect, disinhibition, poor judgment, paranoid delusions, emotional lability, delirium, hallucinations, and catatonia) in patients with CPM without any focal neurological deficits [3–6, 11–13]. Diffusion-weighted MRI (DWI) characteristically shows restricted diffusion in the central pons, and Positron Emission Tomography (PET) scan has been demonstrated to detect central pons hypermetabolism in patients with CPM [14–18].

Here, we describe a case of CPM with delirium and acute psychotic symptoms as the main manifestation of the syndrome, in the absence of focal neurological deficits, with evident hypermetabolism on PET scan, restricted diffusion on DWI MRI sequence, and minimal signal hyperintensity in the central pons on T2-weighted MRI, which became detectable on neuroimaging only after more than a week from the onset of patient's symptoms.

## 2. Case Presentation

A 51-year-old female with only medical history of asthma and smoking presented to the emergency department of an outlying hospital for evaluation of dizziness, frequent falls, diarrhea, and generalized weakness, which started about 1 week prior to admission. Review of symptoms was positive for fatigue, three 10-second long syncopal episodes with rapid return to baseline, urinary frequency, weight loss, and recent upper respiratory tract infection treated with azithromycin. Patient also reported to have had associated generalized weakness, lethargy, ataxia, and slurred speech, but no fever, night sweats, chills, and no history of prior neurological or psychiatric disease.

On further investigation into her history, it was found that, six months ago, the patient had a mammogram revealing a breast mass with inconspicuous weight loss. However, she did not follow up with her referral for an ultrasound.

**Figure 1:**
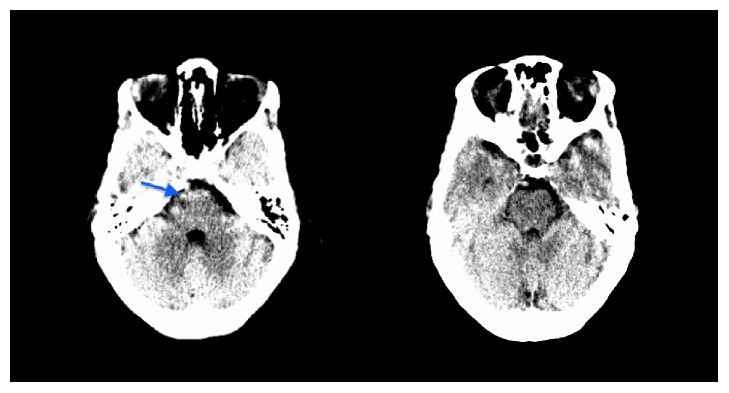
CT scan of the head on admission. Imaging shows no gross acute intracranial abnormality, except for mild bifrontal cerebellar atrophy and a 0.5 × 0.3 cm focal hypodense signal in the right pons (arrow), defined as beam hardening artifact.

Physical examination in the emergency department showed a somnolent and cachectic appearing female, who was nevertheless alert and oriented to time, place, and person. No focal neurological deficits were detected. Laboratory investigations revealed severe hyponatremia (106 mMol/L), hypokalemia (3 mMol/L), and hypochloremia (54 mMol/L). Treatment was initiated by administration of hypertonic saline, subsequently transitioned to normal saline. Chest X-ray and electrocardiogram were unremarkable. Computed tomography (CT) of the head without contrast showed no acute intracranial abnormality except for a small hypodense area seen at the right portion of the pons, attributed to a beam hardening artifact (Figure 1).

**Figure 2:**
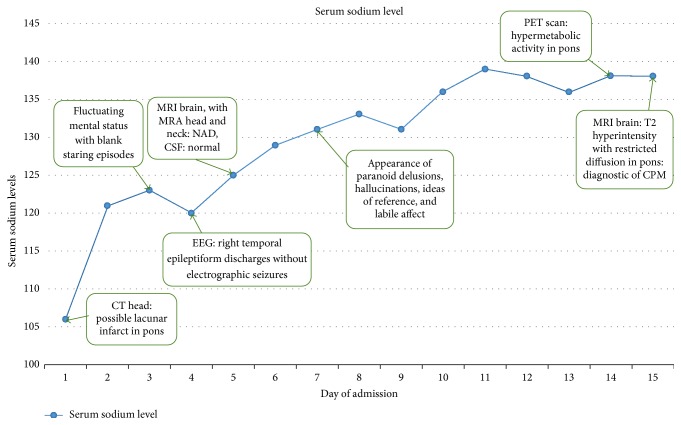
Serum sodium levels (in mMol/L) during hospitalization.

Within 24 hours of admission, the patient's serum sodium was corrected from 106 mMol/L to 121 mMol/L, a 15-point increase (Figure 2). Hypertonic saline was discontinued and replaced with 0.25% normal saline (NS) with 20 mEq KCL to maintain a sodium level in the mid-120s. At 48 hours, patient's sodium level increased to 124 mMol/L. On hospital day 3, she became confused, lethargic, and disorientated to time and place, developed urinary retention, started having intermittent blank stares, and was transferred to our facility for further management. A prompt electroencephalography (EEG) was performed, which showed diffuse background slowing consistent with moderate diffuse cerebral dysfunction, along with infrequent right temporal epileptiform discharges. She was placed on intravenous (IV) fosphenytoin and oral (PO) levetiracetam.

On day 4 of admission, with a sodium level of 120 mMol/L, the patient developed symptoms of acute psychosis: hypervigilance, persistent repetition of words, visual hallucinations, and frenzied speech. Shortly after, she became nonverbal and was unable to follow commands. It was suspected that she could have herpes simplex encephalitis; thus, while screening work-up was initiated, she was started on IV acyclovir.

**Figure 3:**
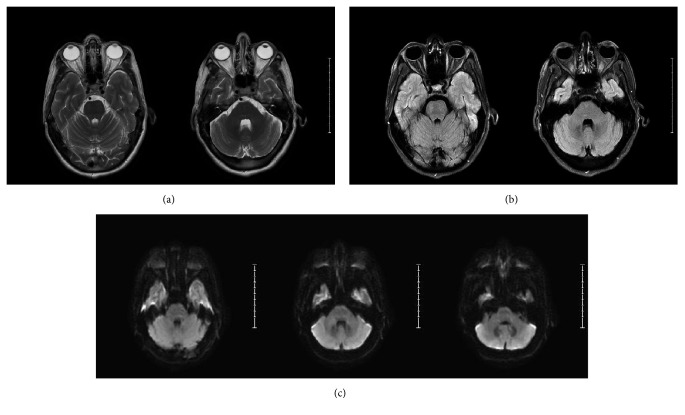
MRI obtained on hospital day 5. T2-weighted images (a), FLAIR (b), and DWI (c) showed no evidence of intracranial pathology.

On day 5, brain MRI was obtained (Figure 3) and lumbar puncture (LP) was performed to evaluate for structural, infectious, inflammatory, demyelinating, vasculitis, and/or autoimmune etiology. Neither neuroimaging nor cerebrospinal fluid (CSF) analysis revealed any significant abnormality. Urine drug screen, thyroid function profile, urinalysis, viral serology, systemic inflammatory and vasculitis markers, and cultures of fluids collected from various sources (blood, urine, and CSF) were all negative. Autoimmune encephalitis panel in blood and CSF also yielded negative results.

On day 7, as sodium level rose to 131 mMol/L, the confusion progressed; the patient was able to state her name but could not follow commands unless repeatedly prompted. She started expressing paranoid delusions, tangential speech, and echolalia. Generalized myoclonic tremor in all extremities was also noted on examination. A repeat EEG was obtained and showed mild nonspecific encephalopathy with no epileptiform activity. Given progression of patient's acute psychotic illness psychiatry consult was requested, which concluded that her behavioral symptoms were not driven by a primary psychiatric disorder but instead could be secondary to a medical illness.

The patient's sodium level rose to 139 mMol/L on hospital day 11. She continued to have visual hallucinations, paranoia, and persecutory delusions. She spoke to the television, had ideas of reference, and identified hospital staff with members of her family. The nonspecific myoclonic tremor in upper extremities continued with no obvious improvement. No other focal deficits were found on neurologic examination.

**Figure 4:**
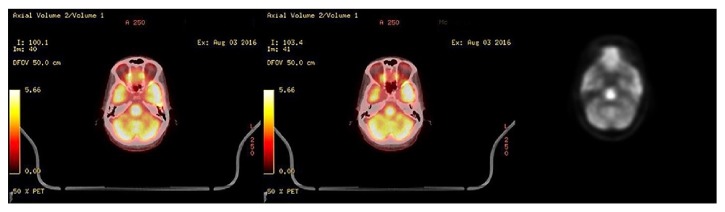
Head PET scan with F-fluorodeoxyglucose tracer on hospital day 14. Scans display focally intense hypermetabolic activity at the pons.

**Figure 5:**
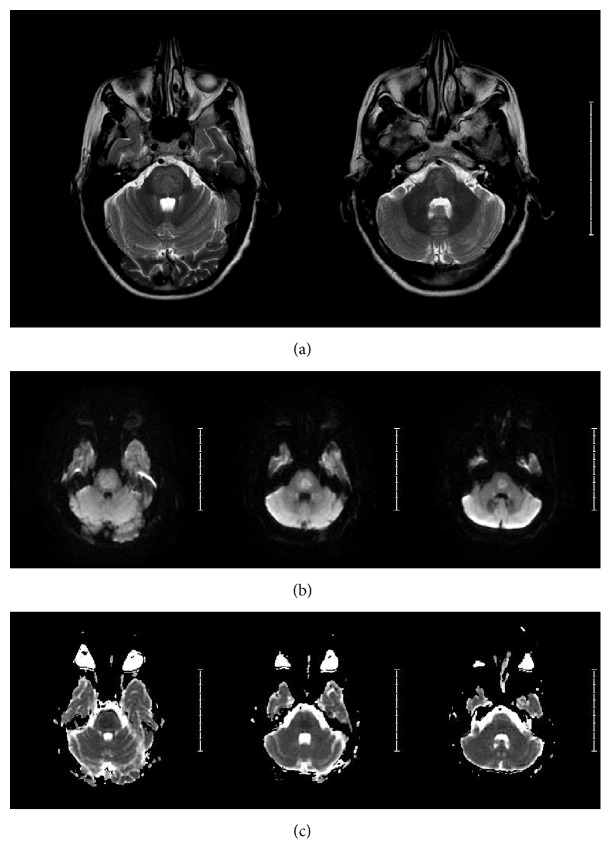
MRI with and without contrast on hospital day 15. T2-weighted MRI shows mild diffuse hyperintense signal changes in the pons (a); DWI sequence shows restricted diffusion (b) with corresponding ADC hypointensity (c) in central pons. These findings were not present when compared to MRI on hospital day 5 (Figure 3) and are consistent with central pontine myelinolysis.

On day 14, extending the diagnostic work-up to include paraneoplastic brain syndrome, a full body PET scan was obtained to assess for occult malignancy, especially considering the known breast mass on previous mammogram, a history of smoking, and elevated serum carcinoembryonic antigen (CEA), obtained during this admission. While skeleton, neck, chest, abdomen, and pelvis scans showed no abnormal uptake of the tracer (data not shown), PET scan revealed abnormal hypermetabolic activity in the pons, suggestive of possible central pontine myelinolysis (Figure 4). Follow-up brain MRI with contrast was then obtained on day 15, which showed restricted diffusion in the central pons confirming the diagnosis of CPM (Figure 5).

The patient's clinical status did not change much with neurological treatment, consisting of a combination of antiepileptic medication, benzodiazepine, and atypical antipsychotic; delirium and hallucinations continued; she was still unable to follow complex commands and properly recognize faces and still had mild generalized myoclonic tremor, while all her metabolic and vital parameters were completely normalized. She was eventually discharged to a skilled nursing facility for further symptomatic management and rehabilitation.

## 3. Discussion

Central pontine myelinolysis (CPM) is a rare neurologic disorder caused by acute demyelination of the central pons. Rapid correction of serum sodium is the most common iatrogenic etiology; others include chronic alcoholism, malnutrition, liver failure, severe burns, and prolonged diuretic use [19]. The most common clinical manifestations of CPM include spastic quadriparesis, dysarthria, pseudobulbar palsy, and altered mental status. These symptoms can be delayed, occurring 2 to 14 days after rapid correction of hyponatremia. In most severe cases, “locked-in syndrome” or even death can occur [20, 21]. Extrapontine myelinolysis (EPM) is a pathophysiologically similar condition affecting 10% of patients with CPM [22]. In EPM, demyelinating lesions occur in brain structures other than the pons, including the basal ganglia (e.g., caudate nucleus), cerebellum, internal capsule, and thalamus. Recent literature has suggested that extrapontine lesions are associated with reversible movement disorders, such as parkinsonism, dystonia, catatonia, mutism, myoclonic jerks, and/or choreoathetosis [2, 9, 11–13]. Both CPM and EPM make up the spectrum of osmotic demyelination syndrome, which affects oligodendrocytes with relative preservation of neuronal axons [9]. The characteristic location of demyelination is thought to occur at the high grey-white matter admixture of the central pons and extrapontine sites in CPM and EPM, respectively.

The work-up of a hyponatremic state initially involves establishing the underlying cause for alteration of sodium metabolism [23]. In our patient, laboratory values for antidiuretic hormone (ADH), urine and serum osmolality, glucose, protein, and lipids were all normal. It is also important to make the distinction between acute and chronic hyponatremia. According to Tzamaloukas et al., while acute hyponatremia (onset less than 48 hours) showed more pronounced brain cell swelling and severity, there is a lower risk of CPM after correction of the sodium level [24]. With chronic hyponatremia, the brain has had the chance to adapt to osmotic fluid shifts; therefore, severe cerebral edema is less likely. However, chronic hyponatremia results in a greater overall loss of brain osmolytes, increasing the risk of CPM after rapid correction of sodium. Unfortunately, it was unknown whether our patient presented to the outlying hospital with acute or chronic hyponatremia.

Our patient's serum sodium level was corrected from 106 mMol/L to 121 mMol/L within the first 24 hours of admission. After 48 hours, the sodium level rose to 124 mMol/L and continued to rise over the following few days to a maximum of 139 mMol/L (Figure 2). According to Martin, a maximum sodium correction of 8 mMol/L within 24 hours, not exceeding 1-2 mMol/L/hour, is recommended [9]. It is now apparent that in our patient the serum sodium level was raised too rapidly during the initial 24 hours, causing her to subsequently develop CPM. To date, once CPM has developed, there are no established effective treatments other than supportive therapy. Thus, prevention is the key to avoid iatrogenic CPM via slow correction of hyponatremia.

Psychiatric and behavioral manifestations are rare clinical presentations in patients with CPM and EPM. The psychiatric disturbances reportedly occur with the common motor manifestations of CPM, such as quadriparesis and dysarthria. The timeline for the onset of psychiatric manifestations in CMP/EPM is a conflict between many authors. Some state that psychiatric disturbances occur within two weeks after the onset of motor symptoms, while others report that motor symptoms can present in the two weeks after initial psychiatric disturbance is observed [3, 4, 11, 25]. To date, the exact pathophysiology of the psychiatric and behavioral manifestations in CPM remains to be elucidated. It is hypothesized that demyelination of the ascending fibers of the reticular activating system (RAS) in the pons is the primary etiology [3, 4]. Disruption of the RAS pathway to the thalamic nuclei may affect several neurotransmitter pathways, resulting in an acute change in behavior. Mostly, all case reports of psychiatric symptoms in CPM have demonstrated additional EPM lesions on MRI (basal ganglia, thalamus, etc.), with the exception of two reports by Price and Mesulam and Walterfang et al. [3, 25].

Our patient developed several manifestations of acute psychotic illness, including confusion, personality change, paranoid hallucinations, tangential speech, echolalia, and ideas of reference, within a week of rapid correction of hyponatremia. The most interesting aspect in our case was the lack of any characteristic motor manifestations of CPM, such as spastic quadriparesis, dysarthria, pseudobulbar palsy, or locked-in syndrome. Our patient displayed some motor signs, such as generalized myoclonic tremor and transient slurred speech with ataxia; however, these are not specific for CPM in the absence of the other characteristic motor manifestations. Thus, our case illustrates that acute psychiatric disturbance in the setting of rapid correction of hyponatremia may be the main presenting symptom of CPM.

The initial brain MRI performed two days after the onset of psychiatric symptoms (five days after rapid correction of sodium) was nondiagnostic and showed no changes in T1-weighted, T2-weighted, diffusion-weighted, or FLAIR image sequences (Figure 3). Generally, the appearance of MRI findings lags the clinical picture and could be delayed up to 10–14 days after rapid correction of sodium [9]. Given primarily neuropsychiatric symptoms in our patient and absence of pontine lesions on imaging, we further investigated other potential causes, including paraneoplastic syndrome secondary to an occult malignancy. As a result of such work-up, the full body PET scan showed increased uptake of radioactive glucose tracer in the pontine region (Figure 4), suggestive of possible CPM. Further assessment of the detected hypermetabolic signal in the pons with repeat MRI confirmed the diagnosis of CPM (Figure 5). It should be noted that neither PET scan nor MRI studies showed any extrapontine involvement in our patient, adding this case report to the few cases of primarily psychiatric manifestations of CPM without EPM.

PET scan is not routinely used for the diagnosis of CPM and the lesion was found incidentally while examining our patient for occult malignancy. The hypermetabolic intensity in the setting of CPM represents increased glucose uptake and metabolism of the phagocytic microglial cells and astrocytes following osmotic demyelination of oligodendrocytes in the pons [26]. It has been reported that the hypermetabolic signal is only transient and later scans may show a hypometabolic focus [17, 18]. This is because oligodendrocytes have limited proliferation capacity and the development of residual gliosis after the initial insult will follow.

The hyperintense signals on T2-weighted MRI and hypointense signals on T1-weighted MRI images in areas of pons demyelination are highly sensitive for diagnosis of CPM. While MRI is helpful in diagnosing CPM, the volume of T2-weighted MRI signal abnormality has been demonstrated to have no association with clinical outcomes in retrospective analysis by Graff-Radford et al. [27].

In our case, a repeat brain MRI was performed during the course of diagnostic investigations to confirm suspected CPM based on PET scan results. The repeat MRI revealed an area of diffusion restriction within the pons on DWI sequence and corresponding hyperintensity on T2-weighted imaging sequence confirming the diagnosis, but without evidence of extrapontine lesions (Figure 5). It should be emphasized that the initial MRI was negative on hospital day 5 and these changes were only noted on day 15, delaying the final diagnosis.

Ruzek et al. have reported the ability of DWI to detect changes before conventional MRI in a patient with suspected CPM within 24 hours of the onset of quadriparesis [15]. Since DWI measures the Brownian motion of water, it is hypothesized that the restricted diffusion in CPM is due to osmotic trapping of water in the intravascular space during the state of relative hypernatremia after rapid correction of hyponatremia [15]. The changes observed on DWI in our case, as well as other case reports, support the diagnostic utility of DWI in detecting acute demyelination in CPM, thus, allowing for prompt diagnosis [14], although the radiographic changes may still lag behind clinical manifestation by several days. However, there is no data in the literature on the validity of brain MRI in early diagnosis of CPM with only neuropsychiatric clinical manifestations in the absence of EPM.

The prognosis of recovery from neurological/neuropsychiatric manifestations of CPM is poor and deficit course is commonly thought to be irreversible. However, several case series and reports have demonstrated that some patients can recover with dependency, independency, or completely [6, 20, 27, 28]. Several case reports have shown reversibility of psychiatric symptoms in CPM with atypical antipsychotic medications [6, 11, 29]; hence, it is recommended that psychiatric manifestations in CPM should be treated with atypical antipsychotics and mood stabilizers, once the patient is neurologically stable. Nevertheless, Vermetten et al. have reported that while significant neurologic deficits may be fully reversible, neuropsychological deficits may remain longer after neurological recovery and may even be permanent [4]. Unfortunately, the psychiatric symptoms in our patient persisted despite optimal treatment with atypical antipsychotics and mood stabilizing therapy.

In conclusion, acute psychosis can present as the main manifestation of CPM. When the history of rapid correction of hyponatremia is present in a patient with neuropsychiatric manifestations, without the characteristic neurological abnormalities frequently seen in CPM, a repeat MRI study within 7–10 days after initial negative imaging could be useful in determining timely diagnosis. DWI seems to be the most specific MRI imaging sequence required for establishing the diagnosis. Currently, the only available treatment of neurological and neuropsychiatric manifestations associated with CPM is symptomatic and many patients do not achieve full recovery. Therefore, prevention is the key to proper management, which should consist of careful and gradual correction of serum sodium level in patients presenting with significant hyponatremia.
